# Phencynonate S-isomer as a eutomer is a novel central anticholinergic drug for anti-motion sickness

**DOI:** 10.1038/s41598-018-38305-9

**Published:** 2019-02-13

**Authors:** Pingxiang Xu, Ying Liu, Liyun Wang, Yi Wu, Xuelin Zhou, Junhai Xiao, Jianquan Zheng, Ming Xue

**Affiliations:** 1 Department of Pharmacology, Beijing Laboratory for Biomedical Detection Technology and Instrument, School of Basic Medical Sciences, Capital Medical University, Beijing, 100069 China; 2 Beijing Institutes of Pharmacology and Toxicology, Beijing, 100850 China; 3 Yanjing Medical College, Capital Medical University, Beijing, 101300 China; 4 Beijing Engineering Research Center for Nervous System Drugs, Beijing, 100053 China

## Abstract

To compare and evaluate the differences of stereoselective activity, the binding affinity, metabolism, transport and molecular docking of phencynonate isomers to muscarinic acetylcholine receptor (mAChR) were investigated in this study. The rotation stimulation and locomotor experiments were used to evaluate anti-motion sickness effects. The competitive affinity with [^3^H]-QNB and molecular docking were used for studying the interactions between the two isomers and mAChR. The stereoselective mechanism of isomers was investigated by incubation with rat liver microsomes, a protein binding assay and membrane permeability assay across a Caco-2 cell monolayer using a chiral column HPLC method. The results indicated that *S*-isomer was more effective against motion sickness and had not anxiogenic action at therapeutic doses. *S*-isomer has the higher affinity and activity for mAChR in cerebral cortex and acted as a competitive mAChR antagonist. The stereoselective elimination of *S*-isomer was primarily affected by CYP1B1 and 17A1 enzymes, resulting in a higher metabolic stability and slower elimination. Phencynonate *S* isomer, as a eutomer and central anticholinergic chiral drug, is a novel anti-motion sickness drug with higher efficacy and lower central side effect. Our data assisted the development of a novel drug and eventual use of *S*-isomer in clinical practice.

## Introduction

Motion sickness can be caused by traveling, such as ship, car, train, air or space motion, and virtual reality immersion like watching the 3D stereoscopic films. It is a common disease in the modern society. Its symptom includes nausea, vomiting, dizziness, ocular ataxia, pallor, cold sweating, deprementia, drowsiness, headache and persistent fatigue^1,2^. The dehydration and electrolyte disturbance were also appeared when emesis was persistent. Severe motion sickness prevents people to conduct marine, aviation, and/or emergency service tasks. It seriously affects tourism and daily operation as well^3^.

Up to date, the precise neurobiological and pathogenic mechanism of motion sickness is not fully understood, and the underlying molecular bases are also ambiguous, but the etiologic theory about cholinergic function of vestibular system that exciting the network of nuclei is generally useful and accepted^4,5^. Among the anti-motion sickness drugs, the anticholinergic agents (e.g. scopolamine) are still the most common drugs used for the prevention and/or treatment of motion sickness. Scopolamine is the most effective prophylactic agent for short (4 to 6-hour) exposures to severe motion, and probably for exposures of up to several days. However, these agents have obvious side effects, such as antagonism on central nervous system (e.g. sedation, drowsiness)^6^, and seriously limit their wide use on people conducting important social activities or precise tasks. Therefore, it is very necessary to discover and develop novel drugs for the prevention and/or treatment of motion sickness.

**Figure 1:**
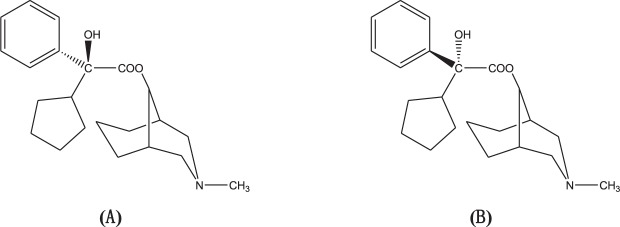
Chemical structures of phencynonate enantiomers, *S*-isomer (**A**) and *R*-isomer (**B**).

Phencynonate {*N*-methyl-9α-(3-azabicyclo [3,3,1] nonanyl)-2′-cyclopentyl-2′-hydroxy-2′-phenylacetate}, a new anticholinergic racemate drug, was synthesized and developed by the Institute of Pharmacology and Toxicology of China^7–9^. Pharmacological evaluation indicated that phencynonate racemate prevents acute motion sickness with a high efficacy similar to that of scopolamine^10,11^. Due to a single chiral carbon in the molecular structure, phencynonate has two optical isomers as *S-* and *R*-isomer configuration^12,13^. The chemical structure of phencynonate isomers is shown in Fig. 1. Phencynonate isomers have been shown distinct difference on *in vivo* pharmacological activities^14^. The stereoselective differences of phencynonate isomers were also investigated in terms of their *in vivo* pharmacokinetic characteristics and their tissue distribution kinetics especially in brain, indicating that there were some marked differences in the main kinetic parameters of *S*- and *R*-phencynonate^15^.

Stereochemistry interactions are the crucial structural characteristics for the active compounds and biological receptor targets. At the molecular pharmacophores, various configurations affect pharmacological and toxicological activities, mainly caused by receptor affinity and activity, drug transport, metabolism and/or pharmacokinetics^16,17^. Therefore, due to the pharmacological, pharmacokinetic, industrial and regulatory implications of stereo-chemical drugs, the significance of stereo-chemistry is paid greater attention^18^. Although the studies have suggested that there are *in vivo* metabolic and active differences existing between *S*- and *R*-phencynonate, up to date, there are no articles about the stereoselective mechanisms on the drug activity for anti-motion sickness, receptor affinity, chiral drug metabolism, transport, protein binding, and molecular docking between phencynonate isomers and mAChR. In the present study, the activity of anti-motion sickness, competitive affinity with binding of [^3^H]-QNB and a molecular docking between the isomers and the mAChR were studied. Based on the chiral HPLC methods, the stereoselective metabolic mechanism of the isomers was also investigated by incubation of the isomers with rat liver microsomes, a protein binding assay and membrane permeability assay across a Caco-2 cell monolayer. The current results showed valuable evidence for the development of a novel chiral eutomer drug for the prevention and/or treatment of motion sickness.

## Results

### Effects of phencynonate *R*- and *S*-isomers on motion sickness

**Figure 2:**
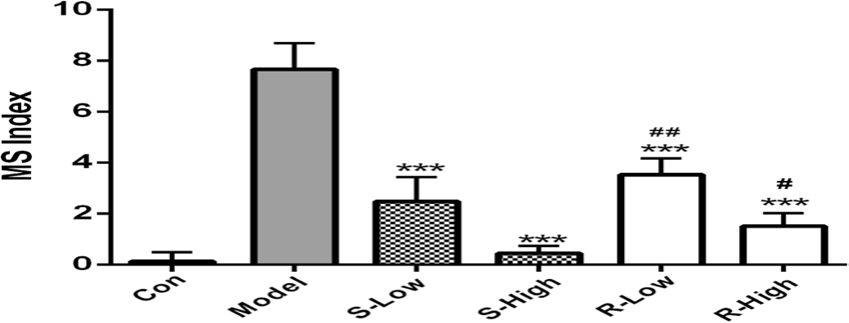
The efficacy comparison of anti-motion-sickness between *R*- and *S*-isomers of phencynonate. n = 10. ^*^p < 0.05 and ^***^p < 0.001 versus the model group, ^#^*p* < 0.05 and ^##^*p* < 0.01 versus the *S*-isomer group. MS index, motion sickness index; S-low, low dose of S-isomer; *S*-High, high dose of S-isomer; R-low, low dose of *R*-isomer; R-High, high dose of *R*-isomer.

To compare the efficacy of anti-motion-sickness of these two isomers, the low and high doses (1.4 and 5.6 mg/kg) of *R*- and *S*-isomers of phencynonate were orally administered to the animals, respectively. The results indicated that both *S*-isomer and *R*-isomer of phencynonate could significantly decrease the index of motion sickness at low or high dose (see Fig. 2). More importantly, *S*-isomer had much higher efficacy of anti-motion-sickness than that of *R*-isomer at the same doses, and there was significant difference on the efficacy against motion sickness between *S*-isomer and *R*-isomer. *S*-isomer at high and low doses significantly reduced the motion sickness index by 93.1% and 72.4%, while *R*-isomer at high and low doses decreased by 79.3% and 53.4%. This suggested that phencynonate *S*-isomer was the more effective drug against motion sickness.

### Effect of the isomers on locomotor activity

**Figure 3:**
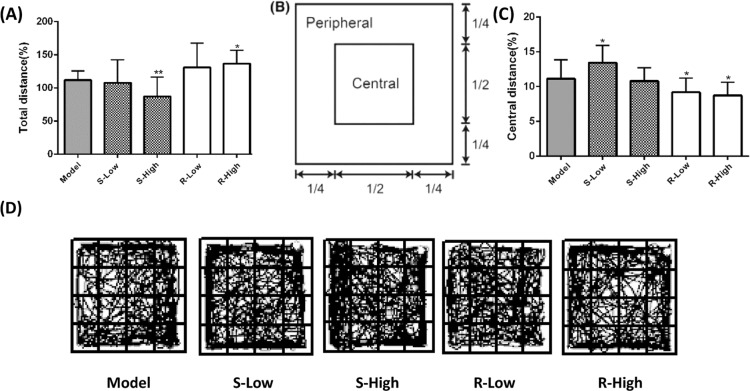
The comparison of locomotor activity between *S*- and *R*-isomers of phencynonate. n = 10. (**A**) The total distance. (**B**) The schematic diagram of arbitrarily divided into central region and peripheral region. (**C**) The central distance ratio. (**D**) The representative locomotor tracks. ^*^p < 0.05 and ^**^p < 0.01 versus the model group. S-low, low dose of *S*-isomer; S-High, high dose of *S*-isomer; R-low, low dose of *R*-isomer; R-High, high dose of *R*-isomer.

Locomotor activity was used for investigating the stimulant or inhibitory effects of chemicals for central nervous system^19,20^. Phencynonate *R*-isomer and *S*-isomer (1.4 and 5.6 mg/kg) were tested on the central nervous system by the locomotor activity test, respectively. The results showed that the *S*-isomer could markedly reduce the total distance (*p* < 0.05, see Fig. 3A), indicating that the *S*-isomer had higher inhibitory efficacy. However, the *R*-isomer reversely increased the total distance, suggesting that this isomer excited the central nervous system and probably had toxic effect at the same doses.

In locomotor experiment, the decrease of central/total ratio or central distance was an important indicator of anxiogenic property^21^. In our study, two doses of *R*-isomer could significantly reduce the central distance ratio for the model group (see Fig. 3C), indicating that *R*-isomer might be anxiogenic effect at these doses. But *S*-isomer did not significantly reduced the central distance ratio for the model group, Fig. 3C), suggesting that the *S*-isomer had no anxiogenic action at the current therapeutic doses.

### Competitive Inhibition for mAChR in Brain

**Figure 4:**
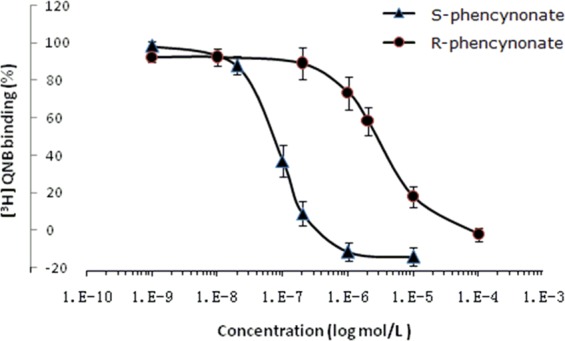
The effects of *S*- and *R*-isomer of phencynonate against the binding of [^3^H]-QNB to the central muscarinic acetylcholine receptors from rat brain.

The binding competitions of *S-*isomer and *R-*isomer with [^3^H]-QNB to mAChR in rat brain homogenate were tested, respectively (shown in Fig. 4). The IC_50_ values of *S-* and *R-*isomer were 8.7 × 10^-8^ M and 2.36 × 10^−6^ M, respectively; and the corresponding K_i_ values of *S-* and *R-*isomer were 46.49 ± 1.27 nM and 1263.12 ± 31.64 nM, respectively. M receptors’ hill coefficients were 1.54 ± 0.06 nH and 1.12 ± 0.03 nH, respectively, indicating that the binding affinity of *S-*isomer to the M receptor was stronger than that of *R-*isomer. Because of the low nH value of *S-*isomer (nH value < 1), the possible allosteric interaction of *S-*isomer to the muscarinic receptor was deduced. Our data indicated that phencynonate isomers acted differently in pharmacological binding to the central M receptors, especially that *S*-isomer had a higher affinity.

### Molecular docking of isomers and m receptor

**Figure 5:**
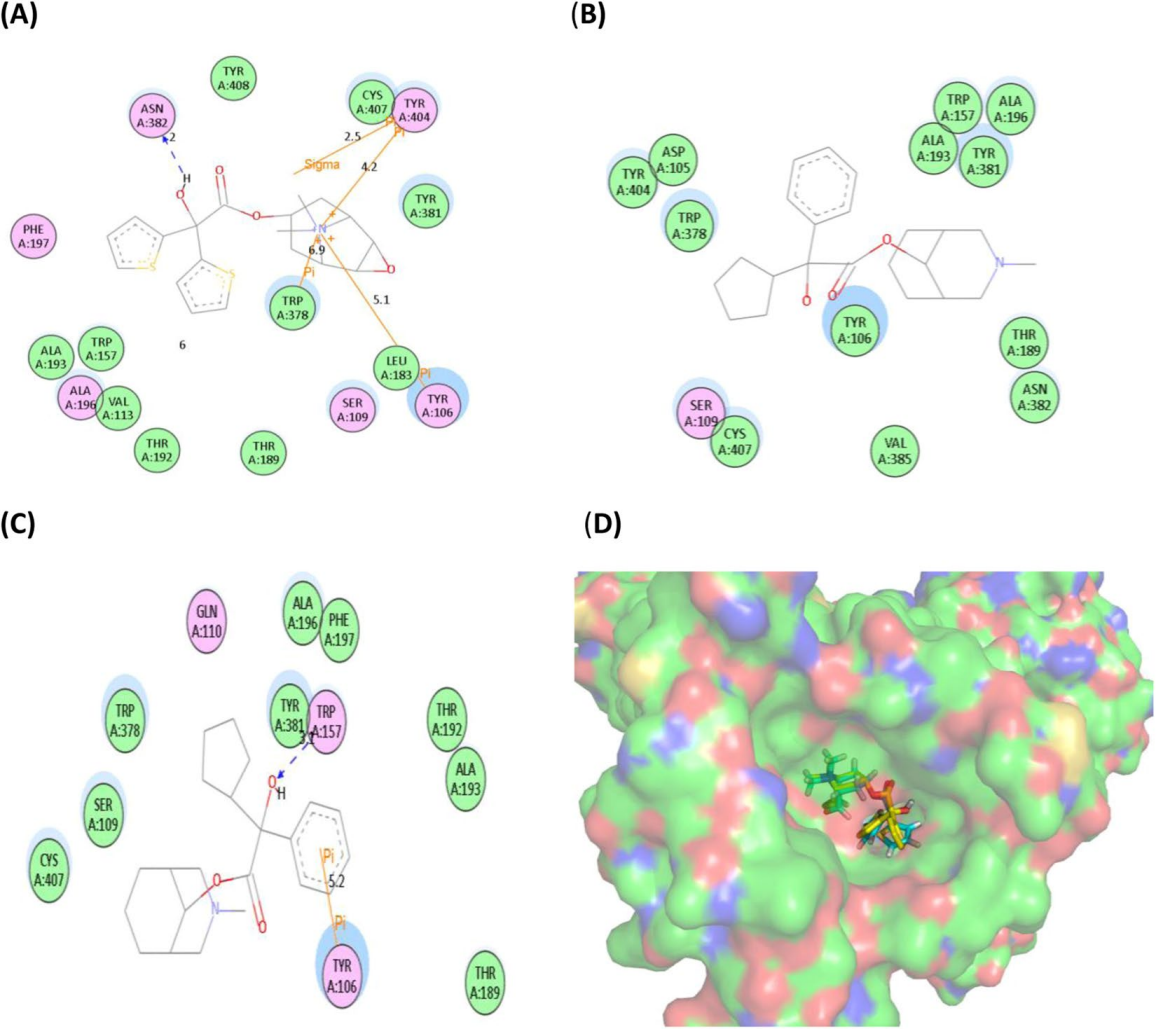
The 2D conformations of Tiotropium (**A**), *S*-isomer (**B**) and *R*-isomer (**C**) of phencynonate interacted with the amino acid residues in the ligand-binding pocket of muscarinic receptor-1 and 3D conformations of Tiotropium (crystal one in yellow sticks and re-docked one in cyan sticks) binding to the ligand-binding pocket of M_1_ receptor (**D**). Colors of residues represent the types of interactions as followed: van der Waals (green), polar (Magenta) and Pi–Pi interaction (yellow). Blue arrows represent the H-bonding with amino acid side chain.

For the validation of docking accuracy, tiotropium was firstly re-docked to the crystallized M_1_ receptor (PBD ID 5CXV). As shown in Fig. 5, the pose of re-docked tiotropium (in cyan sticks) was very close to the original crystallographic one (in yellow sticks) with a RMSD (Root-Mean-Square Deviation) of 1.0 Å which was much smaller than the cutoff of 2 Å, indicating that the parameters used in the current procedures were accurate for molecular docking analysis.

The scores for *S*-isomer and *R*-isomer of phencynonate and tiotropium were 5.02, 3.59 and 5.44, showing that *S*-isomer has stronger binding affinity than *R*-isomer and similar to that of tiotropium (the positive control). As illustrated in Fig. 5A, the quaternary ammonium cation of tiotropium interacted with TYR404, TRP378, and TYR106 through sigma-pi interaction, and its hydroxyl group interacted with ASN382 via hydrogen bond. Figure 5B shows *S*-isomer interacted with TYR106, THR189, ALA193, TYR381 and TRP378 via the van der Waals. While, the hydroxyl group of *R*-isomer interacted with TYR381 and TRP157 via hydrogen bond, and its benzyl group interacted with TYR106 through pi-pi interaction (Fig. 5C).

**Figure 6:**
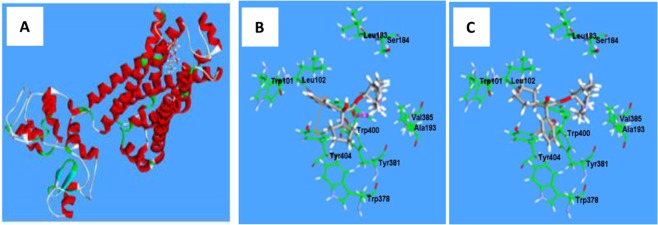
The three dimensional structures of the M_2_ receptor and the action sites of phencynonate (**A**) and the interaction sites between the M_2_ receptor and *S*-isomer (**B**) or *R*-isomer (**C**).

The model structure of the M_2_ receptor protein was based on the seven transmembrane α-helical domains, i.e. heptahelical domains (α1-α7), including the single extracellular ligand-binding domain and the intracellular loop via the α5-α6 helical domains. The hydrophobic cavity of the M_2_ receptor protein was primarily surrounded by the amino acid residue Trp101, Leu102, Tyr106, Phe182, Ser184, Ala193, Trp378, Tyr381, Trp400 and Tyr404 (shown in Fig. 6A). Phencynonate *S* and *R*-isomer were each inserted into the M_2_ receptor. It appeared that phencynonate interacted with the hydrophobic cavity near the extra-membrane via the heptahelical domains. The amino acid residues comprised the active center. The sphere with a radius of 10 Å was defined as the active binding site.

The ligand structures of *S*-isomer and *R*-isomer were built directly using the DS 2.5 software, the 3D structures of the ligands were approximated by the conformation system and optimized, and then the isomer was docked into the defined sphere. The data indicated that there was a marked difference in the mode of action between *S*-isomer and *R*-isomer with M_2_ receptor. The cyclopentane of *S*-isomer was situated deep in the bottom of the active site, resulting in hydrophobic interactions with the Trp378 amino acid residue (Fig. 6B). The bridge ring section of *S*-isomer was bound to the hydrophobic cavity of M_2_ receptor via the hydrophobic interaction. In addition, the bridge ring of *S*-isomer was surrounded by the Leu183, Ser184, Ala193, Val385 and Trp400 residues. The benzene ring of *S*-isomer interacted with the hydrophobic cavity surrounded by the Trp101, Leu102 and Tyr404 residues; at the same time, a π-π interaction was formed with the benzene ring from the Tyr404 residue, stabilizing the complex structure. A stronger hydrogen bond was formed between the carbonyl oxygen in phencynonate molecule and the hydrogen atom from the phenol hydroxyl group of Tyr381 at a distance of 1.88 Å, further stabilizing the complex structure.

The bridge ring section of *R*-isomer was also bound within the hydrophobic cavity of the M_2_ receptor, which was surrounded via the Leu183, Ser184, Ala193, Val385 and Trp400 residues (Fig. 6C). A hydrogen bond was also formed between the carbonyl oxygen in phencynonate and the hydrogen atom in the phenol hydroxyl group of Tyr 381 at a distance of 2.09 Å, helping to stabilize the complex structure. The configurations of *S*-isomer and *R*-isomer were different. The benzene ring of *R*-isomer was situated deep in the bottom of the active site, resulting in a hydrophobic interaction with Trp378. As a result of this interaction, the molecular plane of *R*-isomer was vertical with Tyr404 residue, a π-π interaction between *R*-isomer and Tyr404 benzene ring did not form, and a weak α-π interaction was formed. The hydrophobic interactions between the cyclopentane of *R*-isomer and the M_2_ receptor could result in repositioning of the cyclopentane ring into the hydrophobic cavity surrounded by the Trp101, Leu102 and Tyr404 residues.

From the interaction differences between the *S/R*-isomers and M_2_ receptor and the calculated binding free energy (i.e. *S*-isomer with 47.5 kcal/mol and *R*-isomer with 31.9 kcal/mol), it was qualitatively deduced that *S*-isomer bound tighter to the M_2_ receptor than that of *R*-isomer. These results were also consistent with the observation that *S*-isomer had stronger affinity for the mAChR and a longer half-life^15^.

### Chiral separation and validation

**Figure 7:**
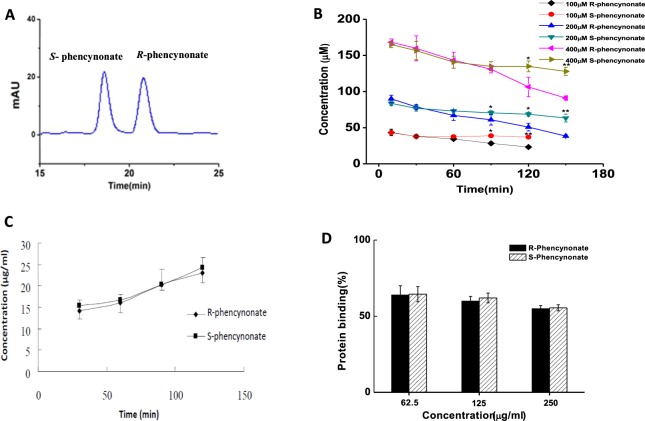
The representative chiral separation of chromatograms for phencynonate racemate (**A**). Comparison of the concentrations of phencynonate enantiomers in rat liver microsomes (n = 5). *p < 0.05 and **p < 0.01 versus *R*-isomer of phencynonate (**B**). The transport concentrations of phencynonate *S*- and *R*-isomers isn Caco-2 cell monolayer (n = 5) (**C**). The proteins binding capacity of phencynonate enantiomers to rat plasma proteins (the initial concentrations of *S*-isomer and *R*-isomer were 62.5, 125 and 250 μg/ml, n = 5) (**D**).

Under the HPLC conditions described above, phencynonate isomers achieved a good baseline separation as shown in Fig. 7A. The elution order of the two isomers was determined by separately injecting the pure *R*- or *S*-isomer. *S*-isomer and *R*-isomer of phencynonate eluted at 19.2 and 21.6 min, respectively. The intra- and inter-day accuracy and precision of the assay were assessed by analyzing the QC samples at three different concentrations for five times on the same day and on three consecutive days, respectively. The precision was calculated as the RSD, and the accuracy was determined as the percentage of deviation between the measured and nominal concentration. Acceptable limits for intra- and inter-day accuracy and precision were set at ±10%. Our data indicated that the validation of detection was reliable and acceptable.

### Chiral metabolism in rat liver microsomes

The results from the *in vitro* biotransformation in rat liver microsomes showed that the unchanged concentrations of *S*- and *R*-isomers were (36.88 ± 0.42) and (23.14 ± 1.86) after 120 min for the low-concentration group (100 μM); (63.33 ± 5.45) and (38.33 ± 1.58) after 150 min for the medium-concentration (200 μM); and (127.93 ± 5.71) and (90.92 ± 2.72) after 150 min for the high-concentration (400 μM). There were significant differences in the metabolic stabilities of the low, medium and high concentrations between *S*- and *R*-isomer (*p* < 0.01; see Fig. 7B). The metabolic rate of *R*-isomer was much faster than that of *S*-isomer, suggesting that hepatic drug enzymes mainly contributed to the stereoselective metabolism, leading to markedly stereoselective differences in pharmacokinetics and brain distribution kinetics^15^.

### Comparison of CYP enzyme protein expression

**Figure 8:**
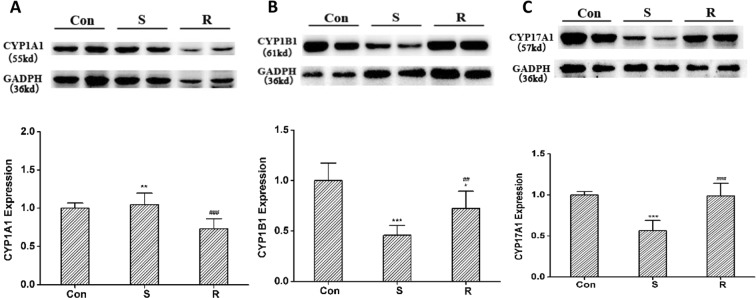
The expression levels of the metabolic enzyme CYP1A1 (**A**), CYP1B1 (**B**) and CYP17 A1 (**C**) in rat liver. Con: the control samples, S: S-phencynonate, R: R-phencynonate. Data represent the mean ± S.D. (n = 3). ^*^p < 0.05, ^***^p < 0.001 versus the control rats, ^###^*p* < 0.001 versus S-phencynonate group.

The CYP enzyme protein levels in rat livers were measured by the Western blot methods. Phencynonate was mainly catalyzed by the metabolic enzyme CYP1A1, CYP1B1 and CYP17A1 in rats. The results indicated that there were significant differences in these CYP enzyme protein levels between *R*- and *S*-isomer of phencynonate. Although *R*-isomer could decrease the extents of CYP1A1 proteins (Fig. 8A), *S*-isomer could markedly decrease the expression levels of CYP1B1 and CYP17A1 proteins, suggesting that *S*-isomer could inhibit CYP1B1 and CYP17A1 enzymes and reduced *S*-isomer *in vivo* metabolism (Fig. 8B,C). Among them, the decreased extents of CYP1B1 and CYP17A1 proteins by *S*-isomer were larger than that of CYP1A1 by *R*-isomer, which were decreased by 52% and 48% via *S*-isomer, and only by 23% via *R*-isomer, respectively, when compared to those of the control group. The data showed that *S*-isomer was the major inhibitor of the metabolic enzyme CYP1B1 and CYP17A1, which mainly affect the metabolic rate of phencynonate in rats. These results interpreted our findings of the differences in drug metabolism and pharmacokinetics of phencynonate in rats^15^.

### Transport across a caco-2 cell monolayer

The membrane permeabilities of phencynonate isomers were investigated using the Caco-2 monolayer cell model. The results showed that *R*- and *S*-isomer have no obvious difference in the permeability within 120 min after dosing (see Fig. 7C), suggesting that the transmembrane transportation both *R*- and *S*-isomer belongs to a first-rate process via the passive diffusion, and there was no stereoselective action in this process.

### Binding of the isomers to plasma protein

The protein binding properties of phencynonate isomers in rat plasma were studied using the equilibrium dialysis. When the concentrations of *R*-isomer and *S*-isomer were 62.5, 125 and 250 μg/ml, the plasma binding rates of *R*-isomer was 64.03%, 60.33% and 54.65%; that of *S*-isomer was 64.46%, 62.18% and 56.02%, respectively (Fig. 7D). The isomers had no significant difference in the protein binding. Within the tested concentration range, the difference between *S*- and *R*-isomer was limited, indicating that the protein binding rate of these two isomers was similar, and had little effect on the stereoselective differences observed for the pharmacokinetics of these two isomers.

## Discussion

Currently, the effective prevention and treatment of motion sickness is still a great challenge in medicine, particularly in aerospace medicine, due to the high incidence and unclear pathogenesis of motion sickness^22^. In clinical practice, only few drugs are suitable for the prevention and treatment of severe motion sickness. The clinical data indicate that the central anticholinergics scopolamine is the most effective and useful single drug against motion sickness, but with prominent unwanted side effects that significantly inhibited the central nervous system and can induce sedation and drowsiness at therapeutic doses^3^. Although many attempts had been made to decrease these side effects, few approaches were considered to be successful and seriously limited the wide use of scopolamine^11^.

The motion sickness occurs with the vestibular system and provocative stimulus as the pre-requisites. The pathogenesis was still not clear and proposed with several hypotheses, such as neurotoxins, neurotransmitter, postural instability, and sensory conflict, etc^23^. These hypotheses could explain some aspects of motion sickness, but the underlying neurobiological mechanism and the precise molecular bases are still ambiguous^24^, requiring the advance of new treatment for motion sickness. Based on the hypothesis that the central cholinolytic activity of anticholinergics may not be parallel completely to their side effects^11^, a series of alicyclic amino alcohol esters were designed, synthesized and evaluated. One of the best compounds, phencynonate and its two isomers were synthesized and discovered by the Institute of Pharmacology and Toxicology of China^7–9^. In animal models, it was demonstrated that the side effects of phencynonate, especially the *S*-isomer, were much milder than those of the central anticholinergic drug scopolamine at equivalent dose of anti-motion sickness^11^. In clinical trials, the overall effective rates for the prevention of seasickness and carsickness of phencynonate (oral 1–2 mg/person) was very significantly higher than that of placebo and control drug. In self controlled rotatory chair experiments, the preventive effects of phencynonate in reducing the changes in electronystagmus and electrogastrogram were statistically significant. Phencynonate could also effectively control the acute attack of vertigo, especially Meniere′s disease and positional vertigo. The side effects of phencynonate were mild dry mouth and drowsiness, and the incidence of drowsiness is significantly lower than that of scopolamine^8,9^.

At least 50% of the drugs used in clinics are the chiral chemicals; and more than 50% of these drugs are used as the racemates in clinical practice, including phencynonate which contains equal proportions of the *R*- and *S*-isomers. According to the information from the Food and Drug Administration of the United States, 45% of the newly approved drugs of molecular entities are optically active, while only 8% are racemates. It shows that there is a crucial tendency, in the last decade, for the development of pure enantiomerical compounds in order to increase treatment effectiveness, decrease the total dose, minimize toxicity and simply define dose-response relationships^25^. Thus, the evaluations of stereoselective properties of chiral compounds are much great important for the development of novel chiral drugs. Future development of drugs with highly selective affinities to the receptor subtypes relevant to motion sickness could produce an anti-motion sickness drug with high efficacy and low side-effects. In our article, the stereoselective properties of phencynonate isomers including the activity, affinity, molecular docking with muscarinic receptor, drug metabolism and transport, were systematically studied.

Firstly, the direct separation and determination of enantiomers by chiral HPLC methods are very important for chiral drug development. The quantitation of enantiomers has also been achieved via the use of an appropriate chiral stationary column or chiral mobile phase conditions. In our work, a chiral HPLC method with a chiral stationary column was developed for rapid quantitative determination of the two isomers of phencynonate racemate. Our results showed that baseline separation was achieved using the β-cyclodextrin chiral stationary phase. This method was validated and could be used for studying the chiral metabolism and transportation of phencynonate racemate.

Binding of [^3^H]-QNB to the M receptors in homogenates of rat brain has a high affinity and specificity, and the characteristics of the binding sites mimic those of central mAChR^26^. The muscarinic antagonists replaced the specific binding sites of [^3^H]-QNB, and nicotinic and non-cholinergic chemicals did but with little affinity, thus, the stereo-selective affinity of phencynonate isomers to mAChR was investigated with [^3^H]-QNB via the sampling of brain membrane receptor. Our results indicated that there were distinct differences in the selective affinity of phencynonate *S*-isomers to mAChR, suggesting that *S*-isomer had the higher pharmacological activities as the CNS-acting drug for preventing acute motion sickness. Based on the animal experiments, the *S*-isomer of phencynonate was the most effective drug against motion sickness and *S*-isomer had no anxiogenic action at the current therapeutic doses.

Molecular mimicry tools can be used to investigate the movement and behavior of the tested molecules, especially the interactions of chiral molecules and receptor proteins^27^. The mAChR model plays a dominant role in drug action and disposition for anticholinergic drugs. The structures of phencynonate isomers bound to the specific target protein provided important information on the interactions between the isomers and mAChR protein, including optimization of protein conformations and isomer position, orientation and active binding site. The present work aimed to study the flexible docking between the mAChR receptor protein and the chiral molecule to predict and reveal the stereoselective interaction between the mAChR and phencynonate isomer, and identify the points of differential binding in the mAChR protein domains between these two isomers. The specific interactions between the isomer and AChR domains indicated that the isomer could be transferred to the target tissue or organ. In our work, the molecular docking was applied to simulate the binding conformations and interactions between phencynonate isomers and M receptor protein. The molecular mimicry results indicated that the bridge ring section of phencynonate *S*-isomer was bound within the hydrophobic cavity of the M receptor via hydrophobic interactions. At the same time, the carbonyl oxygen in the molecule formed strong hydrogen bonds, which further stabilized the whole complex. From the interaction difference between the *S*- or *R*-isomer and the M receptor, and the calculated free energy, we inferred that the *S*-isomer bound to the M receptor better than the *R*-isomer. The results of molecular docking study are consistent with the pharmacodynamic and pharmacokinetic experiments, indicating that *S*-isomer bound better to the mAChR, thus had a more effective action, lower toxicity, longer half-life and slower rate of elimination. The results provide a better understanding of the stereoselective metabolic and active processes, an assist in development of a novel eutomer drug, and its safe and rational use in a clinical setting^28^.

The liver microsomes, plasma protein binding and a Caco-2 cell monolayer were simultaneously used for studying the mechanism of *in vivo* drug disposition differences of the chiral drug phencynonate^29,30^. Our results showed that there were significant differences observed in the metabolic stabilities of the two enantiomers of phencynonate in rat liver microsomes. The hepatic drug enzymes mainly contributed to the stereoselective metabolism of phencynonate *S*- and *R*-isomer, leading to markedly stereoselective differences in pharmacokinetics and brain distribution kinetics. *S*-isomer was the major inhibitor of the metabolic enzyme CYP1B1 and CYP17A1 that mainly affect the drug metabolic rate in rats. The stereoselective elimination of the isomers was primarily affected by liver microsome enzymes, suggesting that *S*-isomers had higher metabolic stability, longer half-life and slower elimination. The levels of transmembrane transportation and plasma protein binding of the two enantiomers were similar, suggesting that limited impact existed on stereoselective pharmacokinetics for phencynonate isomers. These results interpreted our findings of the differences in drug metabolism and pharmacokinetics of phencynonate in rats^15^.

In our current study, phencynonate *S*-isomer has an advantage on the locomotor activity. However, since normal rodents can not vomit, the effects of S-isomer on vomit, another important symptom of motion sickness, can be tested in other species (e.g. dog, cat, ferret, house musk shrew), or other disease rodents models including pica, conditioned taste aversion, conditioned gaping, and hypothermic responses^31^. Besides, this study used the mixture of brain M receptors, thus, further experiments using recombinant proteins of different M receptor subtypes should be carried out to confirm the effects of *S*-isomer on the M receptors subtypes. This is because M3 and M5 receptors in mediating anti-motion sickness actions are more important than M1 or M2, and a role for the M4 receptor in motion sickness is also possible^32–34^.

## Conclusions

This is the first reports that compared and evaluated the stereoselective activity, affinity, metabolism and transportation of phencynonate *S*- and *R*-isomer using the rotation stimulation and locomotor experiment, binding of [^3^H]-QNB to the mAChR, and molecular docking with M receptor, liver microsomes, protein binding and membrane permeability. Our results indicated that *S*-isomer of phencynonate was more effective drug against motion sickness and had no anxiogenic action at therapeutic doses. The affinity from binding with [^3^H]-QNB and the interactions from molecular docking are consistent with those of pharmacokinetics and pharmacodynamics, suggesting that *S*-isomer had stronger affinity and activity for the mAChR. Phencynonate isomers were isocratically separated and identified by the chiral column HPLC method. Our data indicated that there were significant differences in metabolic stabilities of phencynonate isomers in rats, while the transmembrane transportation and plasma protein binding did not affect the stereoselective pharmacokinetics. The stereoselective elimination difference of the isomers was mainly affected via the CYP 1B1 and 17A1 enzymes, resulting in a slower elimination for *S* isomer. In brief, phencynonate *S* isomer, as a eutomer and central anticholinergic chiral drug, has been proved to be a novel anti-motion sickness drug with higher efficacy and lower central side effect.

## Materials and Methods

### Chemicals and reagents

*R*-isomer and *S*-isomer of Phencynonate racemate were synthesized and provided by the Beijing Institute of Pharmacology and Toxicology (Beijing, China). The purities of these three compounds were >99%. [^3^H]-quinuclidinyl benzilate (QNB) was supplied by the Amersham Pharmaceutical Company (Amersham, UK), and atropine sulfate and tiotropium were provided by Sigma Company (St Louis, MO, USA). Triethylamine and methanol were of HPLC grade from the Fisher Scientific (Fair Lawn, NJ, USA). Formic acid (HPLC grade) was supplied by the Dikma Reagent Company (Beijing, China). The other unspecified chemicals, reagents and solvents used were of analytical grade.

### Animals

Adult male Imprinting Control Region ICR mice (body weight 22–25 g) and male Sprague-Dawley rats (100–150 g) were both supplied by the Laboratory Animals Center of Capital Medical University (LAC, CMU, Beijing, China). All animals were randomly divided in the isolated cages, and housed in an air-conditioned room (24 ± 1 °C) under a 12/12-h light/dark schedule. All animals were free access to standard chow and water. The mice were used for the behavioral experiments and the rats were used for the molecular studies. All experiments were performed by following the experimental protocols approved by the Animal Ethnics Committee of Capital Medical University.

### Evaluation of motion sickness severity

Rotation stimulation protocol was performed according to the reference described^35^. The rotation device included a servo-controlled torque motor and six-swing arms (60 cm length) with suspended plexiglas cages in the terminal. Each mouse was enclosed in a cage (the Φ as 20 cm and the H as 25 cm). Sixty mice were orally administered to the tested drugs at 30 min before the stimulation. The device rotated clockwise with a constant angular acceleration (40°/second) until the angular velocity reached 240°/second, and then slowed down^2^. Without pause, the device rotated counterclockwise in a same approach. The rotation stimulation was kept for 40 min. After stimulation, the mice were put on the ground, and the symptoms of motion sickness were scored with the sum of all scores for measuring the severity of motion sickness according to the scoring method listed in Table 1^36^. To avoid habituation effect, all the mice were used only once.

**Table 1:** Evaluation criteria for the index of motion sickness.

Symptoms of motion sickness	Scores
Fecal granules	None: 0; 1 per fecal granule
Urination	None: 0; urination: 1.2
Piloerection	None: 0; light: 0.6; severe: 1.2
Tremor	None: 0; tremor: 1.2

### Determination of locomotor activity

The experiment was performed in a sound-proof room using the video analysis system of mouse spontaneous activity with four video cameras in four chambers (25 × 25 × 31 cm) on the tops^37^ (Shanghai Jiliang Technology Co., Ltd., Shanghai, China). The mice were underwent two pretests for habituation before the formal experiment, which can lower the effect of new environment on locomotor activity. One hundred mice were orally administered to the tested drugs, respectively. After 30 min of drug administration, the mice were placed in the center of the chambers, and their movement was recorded for 30 min. Each chamber was randomly divided into a central region and a peripheral region (see Fig. 2B). To remove the scent clues left by the previous mouse after each testing, the chambers were cleaned with 75% ethanol. The parameters of locomotor tracks were analyzed for central distance, peripheral distance and total travel distance. The ratio of central distance to the total distance was calculated.

### Measurement of competitive inhibition for M receptor

To test the anticholinergic activities of two isomers from phencynonate, the affinities of these compounds were measured by the radio-ligand binding assay with mAChR in rat cerebral cortex^38^. The brains were rapidly removed from the decapitated rats. After excision of the cerebella, each brain was homogenized in ice-cold sucrose (10 volumes, 0.32 M) in a homogenizer at 15,000 rpm and 4 °C for 1 min. The homogenate was centrifuged at 1000 g and 4 °C for 10 min. The pellet, as the crude nuclear fraction, was discarded and the resultant supernatant was centrifuged again at 20,000 g for 30 min. Then, the precipitate was re-suspended in five volumes of Na^+^/K^+^ PBS buffer (50 mM, pH 7.4). The protein concentration was determined by the Lowry method using BSA as the standard, and stored at −80 °C before the [^3^H]-QNB-binding assay.

The reaction contained Na^+^/K^+^ PBS buffer, different concentrations of chemicals, receptor membrane protein (100 μl) and [^3^H]-QNB (6 nM) in 0.5 ml reaction volume. Non-specific binding assay was performed in the presence of atropine sulfate (10 μM). After incubation at 37 °C for 30 min, the reaction was terminated in ice-water bath. The contents were passed through a glass fiber filter (GF/C) using a vacuum. The filter was washed three times with 3 ml of ice-cold buffer under vacuum. Each determination was performed in triplicate with unlabeled QNB to measure the non-specific binding of [^3^H]-QNB. The filters were then placed in vials containing 1 ml of scintillation liquid, and kept for 12 hours. The radioactivity was determined by a liquid scintillation spectrometry (Beckman LS 6500).

Data were analyzed by the curvilinear regression using the GraphPad (ISI, Philadelphia, PA). Each point was plotted as the mean of three independent experiments. In [^3^H]-QNB equilibrium binding assay, the data were fitted to the equation B = B_max_ × F/(K_d_ + F), where B_max_ is the maximum binding capacity, K_d_ is the dissociation constants, and F is the free concentration of the radio-ligand [^3^H]-QNB. In the inhibition assay, the curve was fitted according to a logisteric four parameter function. The IC_50_ values were obtained from at least three independent experiments with 6–8 concentrations of the chemicals. The inhibition constants (K_i_) were calculated using the Cheng-Prusoff equation K_i_ = IC_50_/(1 + L/K_d_), where L and K_d_ are the concentration and the equilibrium dissociation constant of [^3^H]-QNB, respectively.

### Molecular docking experiment

Molecular docking analysis was performed to evaluate the binding affinities and modes of tested compounds by using the Tripos SYBYL-X 2.0 software (St. Louis, MO, USA). The crystal structure of human M_1_ muscarinic acetylcholine receptor with its antagonist tiotropium was obtained from the Protein Data Bank. The size and location of docking site were set with the default values according to those of crystal tiotropium in the receptor. The binding modes of the tested compounds with the highest score in the ligand-binding cavity of M_1_ receptor were chosen for further analysis of docking conformation. The 2D and 3D simulation results were illustrated by the Discovery Studio Visualizer (Accelrys, Inc., CA, USA) and the PyMOL Molecular Graphics System v.1.3 (Schrödinger, LLC, New York City, USA), respectively.

The protein structure of mAChR (M_2_) model was performed via the protein modeling module from the Discovery Studio software/CDOCK protocol. The molecular docking was conducted using the mode of GOLD 4.0.1. The molecular mimicry was completed on the DELL PRECISION PW 690 servers (XEON 5150, 2.66 G, 4 G internal storage). In our study, the sequence of the M_2_ receptor was downloaded from the National Center for Biotechnology Information (NCBI); the ID order is GI 14573539, the reference structure is the Rhodopsin, and the ID is 1U19 on the Protein Data Bank (PDB) based on high structure similarity. The 3D-dimensional structure of the M_2_ receptor protein was built and optimized with kinetic methods using the CHARMm on the DS 2.5. The verification of the structure was conducted via the PROCHECK, indicating that the rational distribution of amino acid residues was similar to the data of GPCR crystal structure published, and the rational structure of the M_2_ receptor protein was finally obtained^39^.

### Chiral HPLC assay

All the analyses were performed using an Agilent HPLC system (Series 1100; Agilent Technology, Palo Alto, USA), comprising a G1322A vacuum degasser, G1310A quaternary pump, G1329A auto sample injector G1316A column thermostat, and G1314AVWD. The chromatography data were recorded and processed with HP Chemstation software (Agilent Technology). The separation was performed on a LiChroCART 250-chiral column (250 mm × 4 mm, i.d. 5 μm). The mobile phase was mixed with methanol and water (60:40, v/v), containing 0.5% (v/v) glacial acetic acid and triethylamine at pH 4.5 condition at a flow rate of 0.3 ml/min. Both phencynonate isomers were measured at 225 nm and the sample injection volume was 20 μl.

### Drug metabolism in rat liver microsomes

Sprague-Dawley rat liver microsomes containing 20 mg/ml of total protein were obtained from the Rild Research Institute (Shanghai, China). The incubation mixture contained 200 μl of phencynonate racemate at various concentrations, 500 μl of rat liver microsomes at a protein concentration of 2 mg/ml, 200 μl of 5 mM NADPH and 100 μl of a 2 mM solution of MgCl_2_. The incubations were carried out with stirring at 37 °C. The final concentrations of phencynonate racemate were 100, 200 and 400 μM.

From 100 μM of phencynonate racemate mixture, 100 μl of the incubation sample was removed after 10, 30, 60, 90 and 120 min. The sampling times were 10, 30, 60, 90, 120, 150 and 180 min in the other two groups. A total of 200 μl of iced methanol was added to stop the incubations immediately upon removing the sample. For the HPLC analysis, the removed sample was vortexed for 1 min, and then centrifuged for 10 min at 10,000 × g. The supernatant was transferred to the HPLC autosampler vials, and directly injected into the HPLC system and analyzed.

### Determination of CYP enzymes by the western blotting

Fifteen rats were randomly divided into three groups, i.e. the control group, *S*-isomer group and *R*-isomer group. The liver microsomes (20 mg per lane) were isolated by 10% sodium dodecyl sulfate polyacrylamide gel electrophoresis (SDS-PAGE), and then transferred to polyvinylidene difluoride membranes, which were blocked at room temperature for 2 h in 5% nonfat milk (dissolved in TBST: trisbuffered saline, with 0.1% Tween 20, pH 7.4). The membranes were then incubated with rabbit polyclonal anti-CYP enzymes or mouse monoclonal anti-GADPH protein (1:10 000) for 1 h at room temperature and overnight at 4 °C. The protein content in supernatants was determined using a BCA protein assay kit (Applygen, China). After being washed three times with TBST (each time for 10 min), the membranes were then probed with secondary antibody conjugated HRP (1:5000) for 1 h at room temperature. The blots were washed three times with TBST, every 10 min, followed by development with ECL plus. The reaction was performed by using ECL detection kit (Applygen, China) in the ChemiDoc MP (Bio-Rad, USA). The Image J (National Institutes of Health, USA) was used to quantify the band intensities.

### Transport across a caco-2 cell monolayer

The Caco-2 cells, originating from a human colorectal carcinoma, were obtained from the American Type Culture Collection (ATCC). Caco-2 cell clone 40 was obtained from the cell culture center of the Institute of Basic Medical Sciences, Chinese Academy of Medical Sciences (IBMS, CAMS, Beijing, China). The cells were cultivated on the millicell culture plate inserts (polycarbonate, 0.4 μm, 12 mm, MilliPore) as the references described^40^. The Caco-2 cells of passage numbers 40–50 were used. The transport studies were carried out with cells cultivated in the culture plate inserts for three weeks. At this time, the Caco-2 cells have differentiated and the fused monolayers of polarized cells have well-developed apical brush borders and the tight junctions with a transepithelial electrical resistance of 600–800 Ω·cm^2^.

The transport experiments were initiated by rinsing the cells twice with PBS carefully. 500 μl of phencynonate racemate was added to the apical side (AP), and 1000 μl of HBSS was added to the basolateral side (BL). 100 μl of sample was collected from the basolateral side and replaced with fresh HBSS at 30, 60, 90, and 120 min, respectively. The removed sample was added to 100 μl of methanol, then vortexed for 1 min and centrifuged for 10 min at 10,000 × g. The supernatant was transferred to the HPLC autosampler vials, and directly injected into the HPLC system and analyzed.

### Plasma protein binding rate in rats

The equilibrium dialysis experiments were performed as described by Duan *et al*.^41^. In this experiment, the racemic phencynonate solution was prepared at 125, 250 and 500 μg/ml in the sample vials. The blank rat plasma sample was first added into a semi-permeable membrane dialysis bag, and then the dialysis bag was submerged into the sample vial. The sample vial was placed in a refrigerator for 48 h at 4 °C. After equilibration, 200 μl of sample from both inside and outside the dialysis bag was removed. 400 μl of methanol was added to the removed samples, vortexed for 1 min, and then centrifuged at 10,000 × g for 10 min. The supernatant was used for the HPLC analysis. The plasma protein binding rate in rats was calculated by the following Eq. (1), in which C_in_ is the concentration inside the dialysis bag; C_out_ is the concentration outside the dialysis bag.

1 $${\rm{Plasma}}\,{\rm{protein}}\,{\rm{binding}}\,{\rm{rate}}=({{\rm{C}}}_{{\rm{in}}}-{{\rm{C}}}_{{\rm{out}}})/{{\rm{C}}}_{{\rm{out}}}\times 100 \% .$$

### Statistical analysis

The investigators were blinded to the grouping when evaluating the severity of motion sickness. Data are presented as the mean ± standard deviation (S.D.). A paired *t* test was used for comparing the data between the *S*- and *R*-isomers of phencynonate. Statistical analysis was performed using ANOVA. The *p* value < 0.05 was considered statistically significant.

## Supplementary information

Dataset 1
