# Supplementary material for: Phencynonate S-isomer as a eutomer is a novel central anticholinergic drug for anti-motion sickness
Source: Sci Rep. 2019 Feb 13;9:2000. doi: 10.1038/s41598-018-38305-9 (PMC6374516; doi:10.1038/s41598-018-38305-9)
Supplement: Supplementary file 1 — Dataset 1 [file 41598_2018_38305_MOESM1_ESM.pdf]

Supplementary information

Phencynonate *S*-isomer as a eutomer is a novel central anticholinergic drug for anti-motion sickness

Pingxiang Xu[1, 3], Ying Liu[1], Liyun Wang[2], Yi Wu[1], Xuelin Zhou[1], Junhai Xiao[2], Jianquan Zheng[2], Ming Xue[1, 3, *]

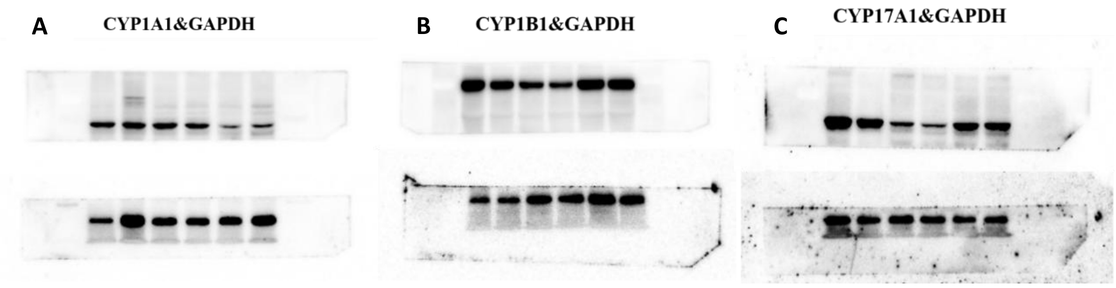

Figure S1 The full-length gels and blots of CYP1A1&GAPDH, CYP1B1&GAPDH and CYP17A1&GAPDH.All of the gels and blots exposure at auto-contrast mode in AIC.AlphaView software.
